# Elucidation of Mechanism of Soil Degradation Caused by Continuous Cropping of *Dictyophora rubrovalvata* Using Metagenomic and Metabolomic Technologies

**DOI:** 10.3390/microorganisms13092186

**Published:** 2025-09-19

**Authors:** Chengrui Lu, Guozheng Qian, Ludi Luo, Yunsong Peng, Hao Ren, Bo Yan, Yongyan Xu

**Affiliations:** Faculty of Landscape Architecture and Horticulture, Southwest Forestry University, Kunming 650224, China; ewewvbqt@protonmail.com (C.L.); 17308747062@163.com (G.Q.); luoludi2018@163.com (L.L.); peng19981204@126.com (Y.P.); renhao19981007@163.com (H.R.); yanbodr@aliyun.com (B.Y.)

**Keywords:** *Dictyophora rubrovalvata*, continuous cropping, metagenome, metabolome

## Abstract

*Dictyophora rubrovalvata* is a soil-cultivated edible fungus with high economic and medicinal value, yet its continuous cultivation is frequently hindered by cropping obstacles. To elucidate the underlying ecological mechanisms, this study employed metagenomic sequencing and untargeted metabolomics (UHPLC–OE–MS) to analyze the changes in soil microbial communities and metabolite profiles under different continuous cropping treatments (CC0: Uncultivated; CC1: one cropping cycle; CC2: two cropping cycle; CC3: three cropping cycle.). Continuous cropping significantly decreased soil pH from 7.94 to 7.52 and available phosphorus (AP) from 213.69 mg/kg to 15.7 mg/kg, while increasing available nitrogen (AN) from 284.5 mg/kg to 886.33 mg/kg. The Shannon index of fungal communities rose from 3.15 to 4.55. Notably, the relative abundance of the beneficial bacterium *Sphingomonas* declined from 15.63% to 1.12%, whereas the pathogenic fungus *Aspergillus* increased from 0.06% to 3.06%. A total of 1408 secondary metabolites were detected, with 39 significantly upregulated and 416 downregulated in CC3 compared to CC0. Several autotoxic compounds, including ferulic acid, hydroxycinnamic acid derivatives, and jasmonic acid, were enriched and positively correlated with pathogenic fungi. These results suggest that continuous cropping may reshape the soil microecosystem by promoting autotoxic metabolite accumulation and pathogenic Microbial enrichment, thereby contributing to soil degradation and cropping obstacles.

## 1. Introduction

**Figure 1:**
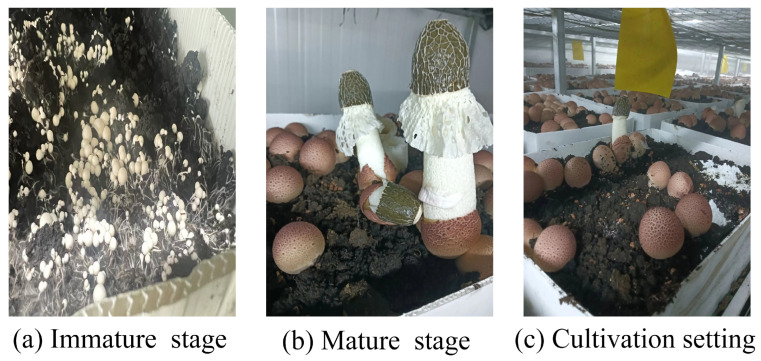
Cultivation and morphological characteristics of *Dictyophora rubrovolvata*.

*Dictyophora rubrovolvata*, classified under Basidiomycota, Gasteromycetes, Phallales, Phallaceae, and Dictyophora [1] (Figure 1), is highly favored by consumers due to its richness in polysaccharides, amino acids, vitamins, flavonoids, and other bioactive compounds [2,3,4]. Yunnan and Guizhou provinces are the main production regions, where the Dictyophora industry plays a significant role in the local specialty economy [5]. As a soil-cultivated edible fungus, *Dictyophora rubrovalvata* requires stimulation from soil cover to initiate fruiting body formation—a unique biological feature that also makes it vulnerable to continuous cropping disorders (CCDs). Long-term monoculture leads to weakened mycelial growth, poor fruiting body development, frequent pest and disease outbreaks, and considerable reductions in both yield and quality, which seriously limit the sustainable development of the industry [6,7,8].

The mechanisms underlying CCDs are complex, involving degradation of soil physicochemical properties, decline in beneficial microbes, enrichment of pathogenic fungi, and accumulation of autotoxic compounds [7]. Studies have shown that continuous monoculture of *Dictyophora rubrovalvata* results in soil acidification and significant reductions in nutrients such as AN, AP, and AK, ultimately lowering yield [9]. Moreover, CCDs are also associated with structural and functional disruptions in the soil microbial community [10,11].

Soil microorganisms play a crucial role in maintaining ecosystem balance, nutrient cycling, and fruiting body induction. The stability of microbial communities directly affects crop health and productivity [12,13,14,15]. A balanced bacterial–fungal ratio in soil enhances system stability and resistance to pathogens. However, continuous cropping often drives a shift from a bacterial-dominant to a fungal-dominant community, and higher fungal diversity and abundance are positively correlated with disease incidence [16,17]. As pathogenic fungi accumulate in the soil, they gain ecological advantages and suppress beneficial or antagonistic microbes that promote fruiting body formation [18,19]. Furthermore, they may secrete specific metabolites or induce the host to release them in ways that support their proliferation and survival, exacerbating CCDs [20,21].

Root exudates are important mediators of crop–soil microbe interactions. These compounds contribute to nutrient uptake, physiological regulation, and environmental adaptation, and they also play a role in the onset of CCDs [21,22,23]. Research shows that edible fungal mycelia can selectively shape microbial communities within their ecological niches [24,25]. These microbes are often attracted by low-molecular-weight compounds such as organic acids and amino acids secreted by the mycelium, which promotes rhizosphere colonization and impacts mushroom productivity [26,27,28]. However, some fungal metabolites have inhibitory effects on fruiting body formation. For instance, volatile organic compounds (VOCs) like 1-octen-3-ol and ethylene produced by Agaricus bisporus during colonization significantly suppress fruiting [29,30]. These autotoxic compounds can accumulate in the soil and impair fungal development while also altering microbial diversity, structure, and function [31,32]. Although evidence links allelopathic substances to crop health, their mechanisms and impact on microbial communities remain unclear.

To date, research on continuous cropping in soil-cultivated edible fungi—especially *Dictyophora rubrovolvata*—is still limited. The unclear mechanisms and lack of effective management strategies underscore the need for more in-depth studies. In this study, we collected soil samples under four cropping conditions (CC0 to CC3) and applied metagenomic sequencing and untargeted metabolomics to evaluate how continuous cropping affects the soil microbiome and metabolome. We further explored the potential interactions between microbial taxa and key metabolites, aiming to provide scientific insight into CCD mechanisms and promote the sustainable development of soil-cultivated edible fungi.

## 2. Materials and Methods

### 2.1. Study Area and Experimental Materials

**Figure 2:**
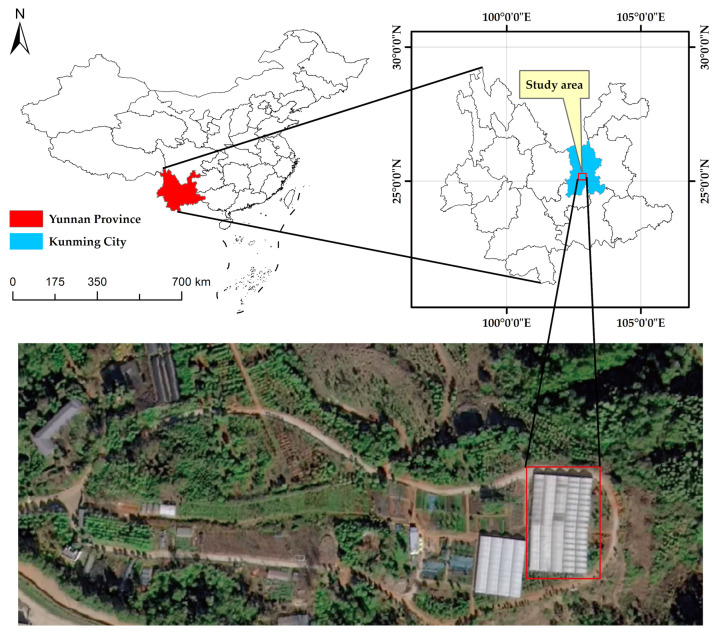
Location map of the study site in the Arboretum of Southwest Forestry University (25°14′ N, 102°45′ E).

The experiment was conducted in a temperature- and humidity-controlled glass greenhouse located in the Arboretum of Southwest Forestry University, Panlong District, Kunming City, Yunnan Province, China (25°14′ N, 102°45′ E). A location map of the study site is provided in Figure 2.

The fungal strain used in this study, *Dictyophora rubrovolvata* ‘Qianyou No.1’, was provided by Guizhou Jinchan Dashan Biotechnology Co., Ltd. (Bijie, China). Surface soil (0–20 cm) was collected from a field that had been left fallow for over five years. After removing visible debris, the soil was passed through a 2 mm sieve, mixed with quicklime at 3% (*w*/*w*), and sealed with plastic film for 60 days under natural conditions to eliminate potential harmful microorganisms. Following treatment, 35 kg of soil was placed into each cultivation box (dimensions: 525 mm × 360 mm × 230 mm).

### 2.2. Experimental Design

A single-factor randomized block design was employed to simulate the continuous cropping process of *Dictyophora rubrovalvata*. Four treatment groups were established: CC0 (uncultivated), CC1 (first cropping cycle), CC2 (second cropping cycle), and CC3 (third cropping cycle), with the soil reused from the previous cycle in each subsequent group. Detailed planting arrangements are provided in Table S1.

Each treatment included nine cultivation boxes to ensure adequate biological replication and statistical reliability. Each cropping cycle lasted 150 days. Throughout the cultivation period, environmental conditions—including air temperature, humidity, and light—were carefully adjusted according to the developmental stage of *Dictyophora rubrovalvata*. These parameters are summarized in Table S2. Temperature and humidity were automatically controlled via a greenhouse environmental monitoring system equipped with real-time sensors and feedback regulation. Light intensity was manually managed using adjustable shading panels and light diffusers.

### 2.3. Soil Sampling

All soil samples were collected in December 2024. A total of 12 samples were obtained, corresponding to four treatment groups (CC0, CC1, CC2, and CC3), with three biological replicates per group. During sampling, a 2.5 cm diameter soil auger sterilized with 75% ethanol was used to collect soil from the 0–15 cm topsoil layer of each cultivation box using the five-point method (four corners and the center). Soil from three cultivation boxes was manually and thoroughly mixed to form one biological replicate, yielding a composite sample of approximately 200 g. To avoid cross-contamination, all sampling tools were autoclaved prior to use and disinfected before each sampling session.

Each composite sample was divided into three parts. Two portions (15 g and 10 g) were placed into sterile 50 mL centrifuge tubes, immediately transferred to a 4 °C ice box for transport, snap-frozen in liquid nitrogen within 30 min of sampling, and stored at –80 °C for microbiome and metabolome analyses. The remaining soil was air-dried at room temperature and used for soil physicochemical property analysis.

### 2.4. Analysis of Soil Physicochemical Properties

Soil samples were naturally air-dried, cleared of stones and plant residues, ground, and sieved through a 2 mm mesh prior to analysis. The soil pH was determined in a 1:2.5 (*w*/*v*) soil-to-water suspension using a calibrated pH meter (NY/T 1377-2007). Total nitrogen (TN) was analyzed using the concentrated sulfuric acid–hydrogen peroxide digestion followed by the Kjeldahl method (NY/T 1121.1-2006). Total phosphorus (TP) was determined using the sulfuric acid–hydrogen peroxide digestion-molybdenum antimony anti-colorimetric method (NY/T 1121.8-2006). Total potassium (TK) was measured by flame photometry after hydrofluoric acid digestion (NY/T 1121.19-2006). Alkali-hydrolyzable nitrogen (AN) was quantified by the alkaline hydrolysis diffusion method using 1 mol/L NaOH (NY/T 1121.7-2006). Soil organic matter (OM) was assessed using the potassium dichromate oxidation–spectrophotometric method (NY/T 1121.6-2006). Available phosphorus (AP) was measured using the sodium bicarbonate extraction–molybdenum antimony colorimetric method (NY/T 1121.8-2006), and available potassium (AK) was determined by flame photometry following neutral ammonium acetate extraction (NY/T 889-2004).

### 2.5. Metagenomic Analysis of Soil Microbiomes

#### 2.5.1. DNA Extraction, Library Construction, and Sequencing

Total genomic DNA was extracted from 0.25 g of soil per sample using the PowerSoil^®^ DNA Isolation Kit (QIAGEN, Germantown, MD, USA), following the manufacturer’s instructions. DNA concentration was measured using a Qubit fluorometer (Invitrogen, Carlsbad, CA, USA), and integrity was assessed by 1% agarose gel electrophoresis. Paired-end libraries were constructed using the TrueLib DNA Library Rapid Prep Kit for Illumina (Ekosai Biotechnology, Beijing, China), including steps of DNA fragmentation, end repair, adapter ligation, PCR amplification, and purification. The library insert size distribution was evaluated using the Agilent 2100 Bioanalyzer (Agilent Technologies, Santa Clara, CA, USA), and final library concentration was measured using a Qubit^®^ 3.0 fluorometer (Life Technologies, Carlsbad, CA, USA). Sequencing was performed on the Illumina NovaSeq 6000 platform (Illumina, San Diego, CA, USA) at Beijing Qingke Biotechnology Co., Ltd. (Beijing, China), generating 150 bp paired-end reads.

#### 2.5.2. Sequence Quality Control and Genome Assembly

Raw sequencing data were quality-filtered using Trimmomatic (version 0.33) to obtain high-quality sequencing data (Clean Tags). The parameters used were as follows: LEADING:3, TRAILING:3, SLIDINGWINDOW:50:20, and MINLEN:100. Metagenomic assembly was performed using MEGAHIT (version 1.1.2) with default parameters, and contig sequences shorter than 300 bp were removed. The assembly results were evaluated using QUAST (version 2.3).

#### 2.5.3. Gene Prediction, Taxonomy, and Functional Annotation

Open reading frames (ORFs) were predicted from the assembled contigs using MetaGeneMark software (version 3.26). The predicted ORFs were translated into amino acid sequences for subsequent construction of a non-redundant gene catalog and functional annotation. All predicted ORFs were clustered using CD-HIT software (version 4.6.6) to generate a non-redundant gene catalog with a sequence identity threshold of 95% and a coverage threshold of 90%. Clean reads were then aligned to the non-redundant gene catalog using Bowtie2 (version 2.2.4) with a minimum alignment identity of 95%. Gene abundance in each sample was calculated using the TPM (Transcripts Per Million) method based on the alignment results.

### 2.6. UHPLC-OE-MS Analysis and Data Processing

A total of 100 mg of soil was weighed into a 2 mL EP tube under low-temperature conditions. Homogenization beads and 500 μL of extraction solution (methanol–acetonitrile–water = 2:2:1, *v*/*v*), containing an isotope-labeled internal standard mixture, were added. Samples were vortexed for 30 s, homogenized at 35 Hz for 4 min, and sonicated in an ice-water bath for 5 min. This cycle was repeated three times. Afterward, the samples were incubated at −40 °C for 1 h, followed by centrifugation at 12,000 rpm (approximately 13,800× *g*, rotor radius 8.6 cm) for 15 min at 4 °C. The supernatant was collected for instrumental analysis.

Non-targeted metabolomic profiling was performed using a Vanquish ultra-high-performance liquid chromatography (UHPLC) system (Thermo Fisher Scientific, Waltham, MA, USA) equipped with a Phenomenex Kinetex C18 column (2.1 mm × 50 mm, 2.6 μm). The mobile phases consisted of water with 0.01% acetic acid (phase A) and a 1:1 (*v*/*v*) mixture of isopropanol and acetonitrile (phase B). The injection volume was 2 μL, and the autosampler was maintained at 4 °C. Mass spectrometry was carried out on an Orbitrap Exploris 120 instrument (Thermo Fisher Scientific, Waltham, MA, USA), with full-scan and MS/MS data acquisition via Xcalibur software (version 4.4). Key MS settings included the following: sheath gas flow rate, 50 Arb; auxiliary gas flow rate, 15 Arb; capillary temperature, 320 °C; full MS resolution, 60,000; MS/MS resolution, 15,000; stepped normalized collision energy (SNCE), 20/30/40; and spray voltage of 3.8 kV (positive mode) and −3.4 kV (negative mode).

Raw MS data were converted to mzXML format using the MSConvert GUI tool in ProteoWizard (version 3.0) and processed on the XCMS online platform (https://xcmsonline.scripps.edu/, accessed on 12 March 2025) for peak picking, noise filtering, and retention time alignment. Quality-controlled data were annotated using the CAMERA software (version 1.46.0) to identify adducts, isotopes, and fragment ions. Metabolite identification was then performed using metaX software (version 1.4.5) in combination with the HMDB (https://hmdb.ca/, accessed on 12 March 2025) and KEGG (https://www.kegg.jp/, accessed on 12 March 2025) databases, generating a metabolite abundance matrix for each sample.

### 2.7. Correlation Analysis Between Dominant Microbial Genera and Differential Metabolites

To explore the potential associations between microbial taxa and differential metabolites, Spearman correlation analysis (|r| > 0.8, *p* < 0.05) was conducted. First, significantly enriched differential metabolites involved in key metabolic pathways were identified separately for each comparison group (CC0 vs. CC1, CC0 vs. CC2, and CC0 vs. CC3). These metabolites were then correlated with the top 20 bacterial and fungal genera (based on relative abundance) from the corresponding treatment groups (CC1, CC2, and CC3). Finally, correlation networks were constructed to visually represent the potential interactions between microbial genera and metabolites under different continuous cropping treatments.

### 2.8. Bioinformatics and Statistical Analysis

The structure of microbial communities was analyzed based on relative abundances at different taxonomic levels. Alpha diversity indices, including Shannon and Simpson, were calculated to assess species richness and evenness [33]. The top 10 phyla and top 20 genera with the highest relative abundances were selected, and stacked bar plots were used to visualize taxonomic composition and variation across samples. Beta diversity was assessed using principal coordinate analysis (PCoA) based on Bray–Curtis dissimilarity, implemented with the “amplicon” and “phyloseq” packages in R [34]. To identify significantly different taxa among treatments, linear discriminant analysis effect size (LEfSe) was applied with LDA ≥ 3 and *p* < 0.05, and the results were visualized by LDA score bar plots [35]. Microbial co-occurrence networks were constructed based on Spearman correlation coefficients (|r| > 0.8, *p* < 0.05) [36] using the “igraph” and “Hmisc” R packages and visualized in Gephi (version 0.10.1) [37,38]. Bacterial and fungal genera with average relative abundances greater than 0.1% within each treatment were included in the network. Network topological parameters, such as average degree, modularity, and clustering coefficient, were calculated, and the top five genera with the highest betweenness centrality were identified as core taxa.

Principal component analysis (PCA) and orthogonal partial least squares discriminant analysis (OPLS-DA) were performed using the “factoextra,” “ropls,” and “ggplot2” packages in R to evaluate sample clustering and group separation [39]. Differential metabolites were screened based on fold change (FC ≥ 1.2 or ≤ 0.833), variable importance in projection (VIP ≥ 1.0) from OPLS-DA, and Student’s *t*-test (*p* < 0.05). Volcano plots were generated using “ggplot2” to visualize metabolite distribution. Pathway enrichment analysis was conducted based on KEGG annotations using hypergeometric testing (*p* < 0.05), and results were visualized with the “clusterProfiler” and “enrichplot” R packages [40].

All statistical analyses were performed using SPSS version 27.0 (IBM, Armonk, NY, USA). Data were first compiled and organized using Microsoft Excel 2021. One-way analysis of variance (ANOVA), followed by Tukey’s Honestly Significant Difference (HSD) test, was conducted to evaluate differences in soil physicochemical properties (pH, TN, TP, TK, OM, AN, AP, and AK) and microbial alpha diversity indices across treatments. Prior to ANOVA, data normality and homogeneity of variance were assessed using the Shapiro–Wilk and Levene’s tests. A significance threshold of *p* < 0.05 was applied. All figures were created and assembled using Adobe Illustrator 2025.

## 3. Results

### 3.1. Soil Physicochemical Properties

As shown in Table 1, continuous cultivation of *Dictyophora rubrovolvata* significantly impacted soil physicochemical properties. With the increase in cropping cycles, soil pH, AP, and TK content decreased significantly. Specifically, the pH values in CC2 and CC3 were significantly lower than those in CC0 and CC1 (*p* < 0.05). The TK content also decreased significantly, from 22.63 ± 1.35 g/kg in CC0 to 4.48 ± 0.77 g/kg in CC3, indicating considerable potassium depletion during the cultivation process. In contrast, AN peaked in CC2 (1055 ± 51.51 mg/kg), exceeding the levels in both CC0 and CC1. AK content increased with successive cropping, reaching 693 ± 51.22 mg/kg in CC3, significantly higher than 484.31 ± 21.81 mg/kg in CC0. However, no significant differences were observed in OM, TN, and TP among the treatments (*p* > 0.05). These changes may be attributed to the selective utilization and degradation of specific nutrient components in the cultivation substrate by fungal mycelia, as well as the progressive accumulation of certain nutrients during the continuous cropping process.

### 3.2. Soil Microbial Diversity

**Figure 3:**
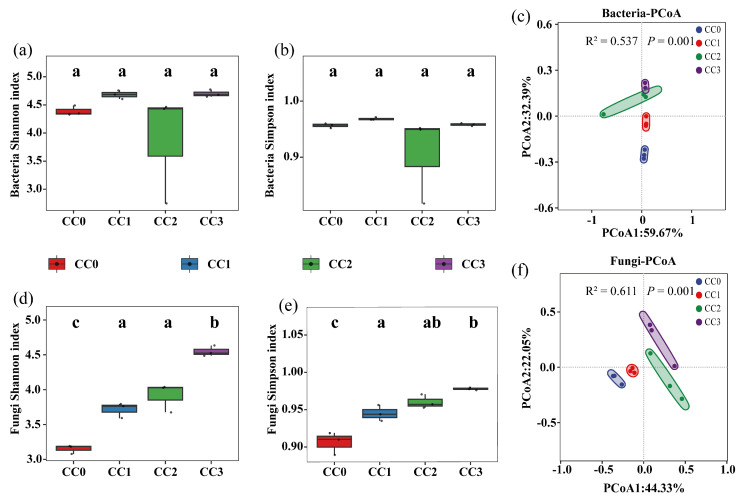
Diversity of soil microbial communities under different continuous cropping treatments, based on Shannon and Simpson indices and PCoA analysis. (**a**,**b**,**d**,**e**) Shannon and Simpson indices for bacterial (**a**–**c**) and fungal (**d**–**f**) communities. (**c**,**f**) Principal coordinate analysis (PCoA) of bacterial and fungal communities. Note: Different lowercase letters above the boxes indicate significant differences among treatments (*p* < 0.05). CC0: Uncultivated; CC1: one cropping cycle; CC2: two cropping cycle; CC3: three cropping cycle.

The alpha diversity of soil microbial communities exhibited distinct patterns under different continuous cropping treatments. For bacteria (Figure 3a,b), there were no significant differences in either the Shannon or Simpson indices among the four treatments, indicating that bacterial richness and evenness remained relatively stable during continuous cropping. The average bacterial Shannon index values for CC0, CC1, CC2, and CC3 were 4.389 ± 0.089, 4.683 ± 0.077, 3.880 ± 0.982, and 4.701 ± 0.065, respectively. The corresponding Simpson indices were 0.956 ± 0.004, 0.968 ± 0.003, 0.906 ± 0.077, and 0.958 ± 0.003.

In contrast, fungal diversity significantly increased with successive cropping cycles (Figure 3d,e). The Shannon index in CC2 and CC3 was significantly higher than in CC0 (*p* < 0.05), with a similar pattern observed for the Simpson index. Specifically, the fungal Shannon index increased from 3.151 ± 0.064 in CC0 to 4.548 ± 0.075 in CC3, while the Simpson index rose from 0.906 ± 0.015 to 0.978 ± 0.002. These results suggest that continuous cropping may promote the proliferation of certain fungal taxa, potentially contributing to the emergence of continuous cropping obstacles.

Principal coordinate analysis (PCoA) based on Bray–Curtis distances (Figure 3c,f) revealed distinct separation of microbial community structures across treatments. This was further supported by PERMANOVA results, which showed significant effects of continuous cropping on both bacterial (R^2^ = 0.537, *p* < 0.001) and fungal (R^2^ = 0.611, *p* < 0.001) communities, with the fungal community exhibiting greater sensitivity to continuous cropping disturbances.

### 3.3. Soil Microbial Composition and Structure

**Figure 4:**
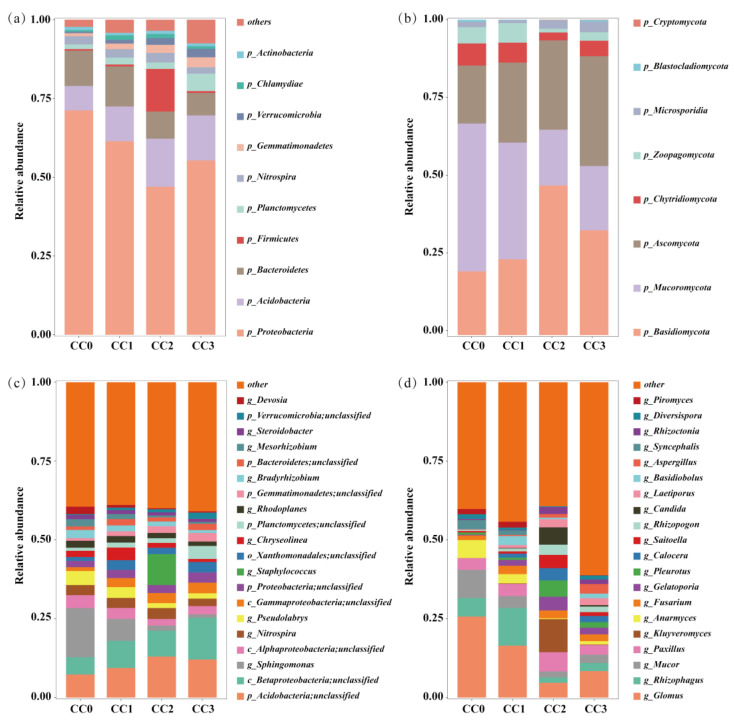
Composition of soil microbial communities under different continuous cropping treatments. (**a**,**b**) Relative abundances at the phylum level and (**c**,**d**) at the genus level. (**a**,**c**) Bacteria. (**b**,**d**) Fungi. CC0: Uncultivated; CC1: one cropping cycle; CC2: two cropping cycle; CC3: three cropping cycle.

Continuous cropping markedly altered the composition and structure of the soil microbial communities. In the bacterial community, *Proteobacteria*, *Acidobacteria*, and *Bacteroidetes* were the dominant phyla (Figure 4a). The relative abundance of *Proteobacteria* decreased from 71.23% in CC0 to 46.98% in CC2, with a slight rebound to 55.33% in CC3. *Bacteroidetes* also declined from 11.21% to 7.12%, whereas *Acidobacteria* increased, reaching a peak of 15.24% in CC2. Additionally, the relative abundances of *Gemmatimonadetes*, *Planctomycetes*, and *Verrucomicrobia* gradually increased with the number of cropping cycles. Fungal communities were mainly composed of *Basidiomycota*, *Mucoromycota*, and *Ascomycota* (Figure 4b). Continuous cropping led to notable increases in *Basidiomycota* and *Ascomycota*, rising from 20.19% and 18.40% in CC0 to 47.41% and 34.83% in CC2, respectively. In contrast, *Mucoromycota* declined sharply from 46.86% in CC0 to 17.68% in CC2.

At the genus level (Figure 4c,d), beneficial bacterial genera such as *Sphingomonas*, *Bradyrhizobium*, and *Devosia* exhibited a decreasing trend with continuous cropping—for instance, *Sphingomonas* declined from 15.63% in CC0 to 1.12% in CC3. Similarly, beneficial fungal genera including *Glomus*, *Rhizophagus*, and *Anaeromyces* showed reduced relative abundances. Conversely, potentially pathogenic fungi such as *Fusarium*, *Aspergillus*, and *Rhizoctonia* increased in abundance, with *Aspergillus* rising significantly from 0.06% to 3.06%. These changes suggest that continuous cropping reduces the abundance of beneficial microbes while enriching potential pathogens, likely contributing to imbalances in the soil microbial ecosystem.

### 3.4. Microbial Networks and Key Biological Biomarkers

**Figure 5:**
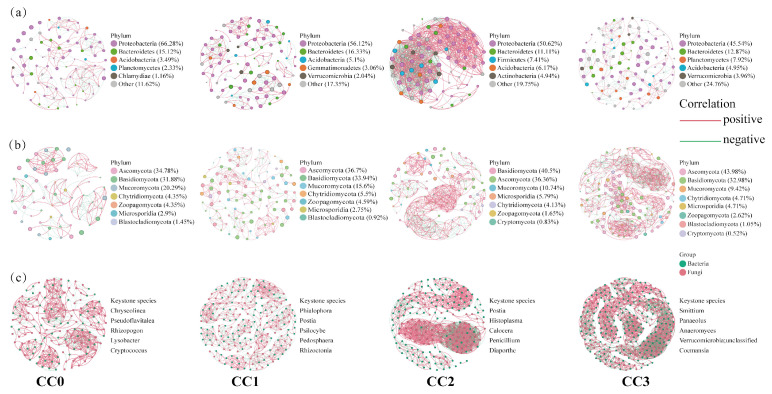
Co-occurrence networks of (**a**) bacterial communities, (**b**) fungal communities, and (**c**) bacterial and fungal communities under different continuous cropping treatments. Red line: positive correlation; green line: negative correlation. For each treatment, five keystone genera with the highest betweenness centrality scores were identified. CC0: Uncultivated; CC1: one cropping cycle; CC2: two cropping cycle; CC3: three cropping cycle.

Compared with uncultivated soil, continuous cropping significantly increased the complexity of soil microbial co-occurrence networks (Figure 5). In bacterial co-occurrence networks, the number of nodes and edges under each treatment were as follows: CC0 (86 nodes, 220 edges), CC1 (98, 233), CC2 (81, 965), and CC3 (101, 253). In fungal networks, the corresponding values were as follows: CC0 (69, 177), CC1 (109, 295), CC2 (121, 586), and CC3 (191, 1135). In the combined bacteria–fungi networks, both connectivity and complexity showed continuous enhancement across treatments: CC0 (155 nodes, 755 edges), CC1 (207, 1407), CC2 (202, 1966), and CC3 (292, 2434). These results indicate that continuous cropping not only strengthens microbial interactions but also promotes the structural complexity of the microbial community. In addition, based on betweenness centrality analysis, the top five keystone genera in each bacteria–fungi network were identified, reflecting dynamic shifts in dominant microbial taxa during the continuous cropping process.

LEfSe analysis (LDA > 3) further revealed significant microbial biomarkers among treatments (Figures S1 and S2, Table S3). In CC0, beneficial bacterial genera such as *Sphingomonas* (*p* = 0.02), *Pseudolabrys* (*p* = 0.03), and *Niastella* (*p* = 0.02), along with symbiotic fungi including *Glomus* (*p* = 0.04), *Anaeromyces* (*p* = 0.02), and *Syncephalis* (*p* = 0.02), were significantly enriched. In contrast, CC3 showed a clear enrichment of pathogenic fungi closely related to edible mushroom diseases, such as *Aspergillus* (*p* = 0.02) and *Trichoderma* (*p* = 0.03). These results suggest that the continuous cultivation of *Dictyophora rubrovolvata* may promote the accumulation of pathogenic fungi in soil, thereby affecting the stability of the soil micro-ecosystem.

### 3.5. Metabolomic Analysis of Soils Under Different Continuous Cropping Treatments

**Figure 6:**
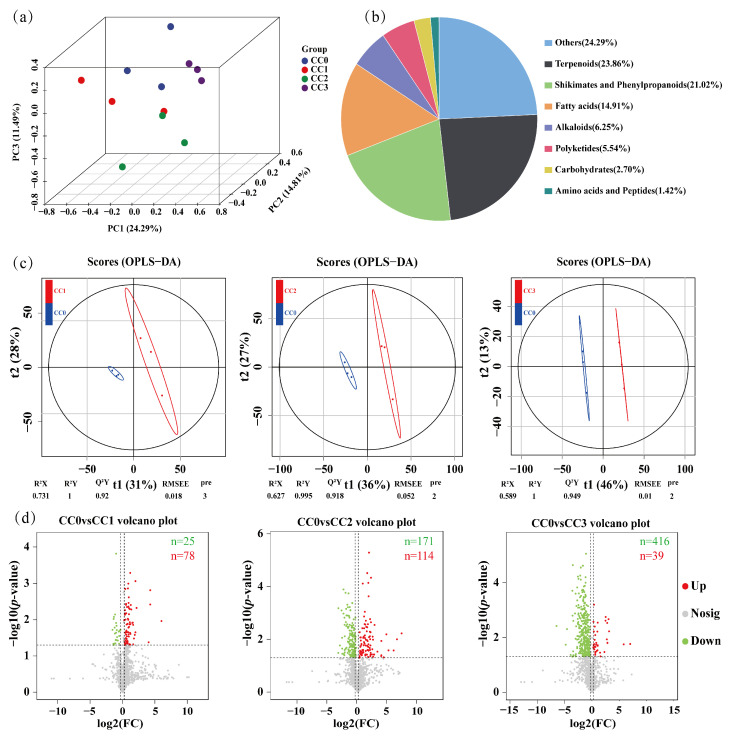
Metabolomic analysis of soils under different continuous cropping treatments. (**a**) Principal component analysis (PCA) of soil metabolites. (**b**) Classification of all identified secondary metabolites based on the Human Metabolome Database (HMDB). (**c**) Orthogonal partial least squares discriminant analysis (OPLS–DA) score plots showing separation between the control group (CC0) and treatment groups CC1, CC2, and CC3. (**d**) Volcano plots of differential metabolites between CC0 and the three treatment groups. Red dots: up-regulated metabolites; green dots: down-regulated metabolites; and gray dots: no differential metabolites. CC0: Uncultivated; CC1: one cropping cycle; CC2: two cropping cycle; CC3: three cropping cycle.

To comprehensively evaluate the impact of continuous cropping of *Dictyophora rubrovolvata* on soil metabolites, an untargeted metabolomic analysis was conducted on 12 soil samples using ultra-high-performance liquid chromatography coupled with Orbitrap Exploris mass spectrometry (UHPLC–OE–MS). A total of 24,993 metabolic features were detected, among which 1408 secondary metabolites were retained after filtering. According to the Human Metabolome Database (HMDB), these metabolites were mainly classified into the following categories: terpenoids (23.86%), shikimates and phenylpropanoids (21.02%), fatty acids (14.91%), alkaloids (6.25%), polyketides (5.54%), carbohydrates (2.70%), and amino acids and peptides (1.42%) (Figure 6b).

Principal component analysis (PCA) revealed clear separations among the different continuous cropping treatments, indicating that continuous cultivation significantly altered the composition of soil metabolites (Figure 6a). This trend was further validated using orthogonal partial least squares discriminant analysis (OPLS–DA), which showed distinct separation between the control group (CC0) and each of the continuous cropping groups (CC1, CC2, and CC3). The OPLS–DA models exhibited good fitness and predictive ability, with R^2^Y/Q^2^Y values of 1/0.92 (CC0 vs. CC1), 0.995/0.918 (CC0 vs. CC2), and 1/0.949 (CC0 vs. CC3) (Figure 6c).

Differential metabolites were screened based on the criteria of VIP > 1, fold change ≥1.2 or ≤0.833, and *p* < 0.05. Volcano plot analysis showed that the number of significantly downregulated metabolites gradually increased with continuous cropping duration, with 25 identified in CC0 vs. CC1, 171 in CC0 vs. CC2, and 416 in CC0 vs. CC3. In contrast, the number of upregulated metabolites showed a rise-then-fall trend, with 78, 114, and 39 detected, respectively (Figure 6d). These results indicate that continuous cropping has a significant impact on the soil metabolome.

### 3.6. KEGG Enrichment of Differential Metabolites Under Continuous Cropping Treatments

**Figure 7:**
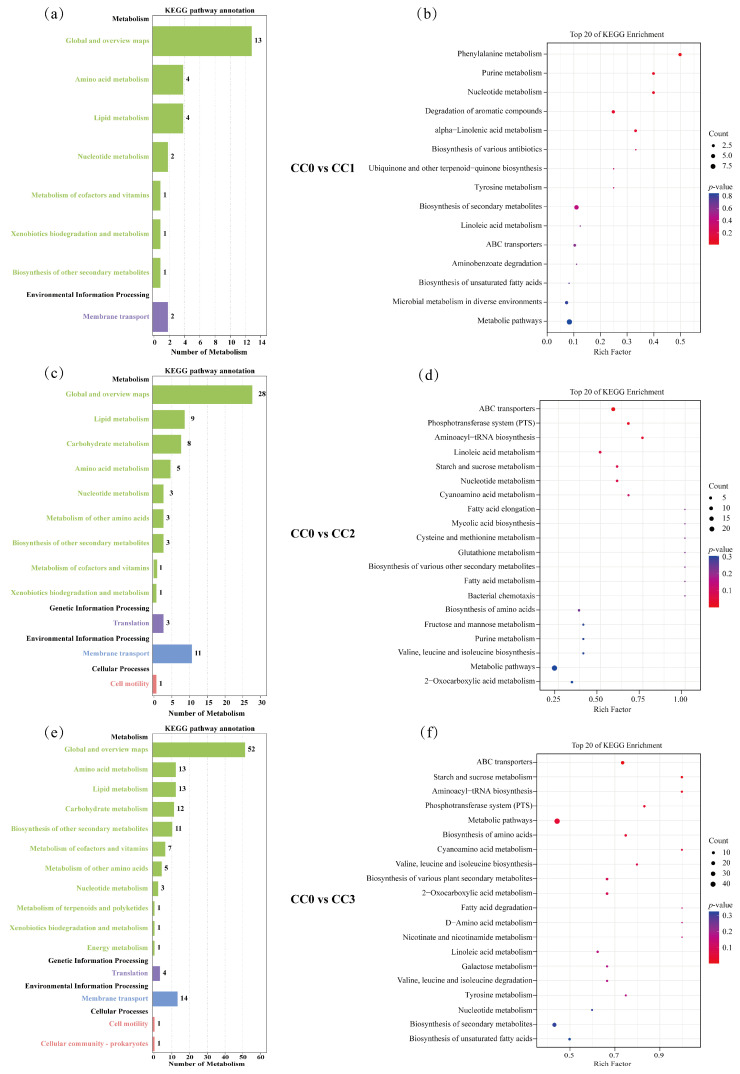
KEGG enrichment analysis of differential metabolites. (**a**,**b**) Differential metabolic pathway classification and KEGG enrichment analysis for CC0 vs. CC1; (**c**,**d**) for CC0 vs. CC2; and (**e**,**f**) for CC0 vs. CC3. In the bar charts (**a**,**c**,**e**), the vertical axis represents KEGG classifications, with Class A categories shown in black and Class B categories in colored text. The horizontal axis indicates the number of metabolites annotated under each Class B pathway. In the bubble plots (**b**,**d**,**f**), the vertical axis lists pathway names, and the horizontal axis indicates the Rich Factor. Bubble color corresponds to *p*-value (darker red indicates more significant enrichment), while bubble size reflects the number of enriched differential metabolites. CC0: Uncultivated; CC1: one cropping cycle; CC2: two cropping cycle; CC3: three cropping cycle.

In the CC0 vs. CC1 comparison group (Figure 7a,b), the differential metabolites were mainly enriched in two KEGG level-1 categories: Metabolism and Environmental Information Processing, involving eight KEGG level-2 subcategories, including Global and overview maps (13 metabolites), Amino acid metabolism (4), Lipid metabolism (4), Nucleotide metabolism (2), Metabolism of cofactors and vitamins (1), Xenobiotics biodegradation and metabolism (1), Biosynthesis of other secondary metabolites (1), and Membrane transport (2), with a total of 15 enriched KEGG pathways. The KEGG enrichment results (Figure 7b) further showed that the significantly enriched pathways were mainly associated with the biosynthesis or degradation of fatty acids, terpenoids, alkaloids, and cinnamic acid derivatives. Notably, some of these compounds (e.g., alkaloids and cinnamic acid derivatives) have been reported to possess antifungal or allelopathic effects, suggesting that they may play an inhibitory role on certain microbial groups during the early stages of continuous cropping.

In the CC0 vs. CC2 comparison group (Figure 7c,d), the enriched pathways spanned four KEGG level-1 categories: Metabolism, Genetic Information Processing, Environmental Information Processing, and Cellular Processes, involving 12 subcategories, including Global and overview maps (28), Lipid metabolism (9), Carbohydrate metabolism (8), Amino acid metabolism (5), Nucleotide metabolism (3), Metabolism of other amino acids (3), Biosynthesis of other secondary metabolites (3), Metabolism of cofactors and vitamins (1), Xenobiotics biodegradation and metabolism (1), Translation (3), Membrane transport (11), and Cell motility (1), with a total of 40 enriched pathways. Among the top 20 enriched pathways (Figure 7d), most were related to the biosynthesis and degradation of alkaloids, fatty acids, amino acids, peptides, carbohydrates, and coumarins. These metabolic changes may contribute to the accumulation of bioactive compounds in the soil, which in turn could interfere with fungal growth or disrupt the balance of rhizosphere microbial communities.

In the CC0 vs. CC3 comparison group (Figure 7e,f), the differential metabolites were similarly enriched in the same four KEGG level-1 categories, involving 15 subcategories, including Global and overview maps (52), Amino acid metabolism (13), Lipid metabolism (13), Carbohydrate metabolism (12), Biosynthesis of other secondary metabolites (11), Metabolism of cofactors and vitamins (7), Metabolism of other amino acids (5), Nucleotide metabolism (3), Metabolism of terpenoids and polyketides (1), Xenobiotics biodegradation and metabolism (1), Energy metabolism (1), Translation (4), Membrane transport (14), Cell motility (1), and Cellular community—prokaryotes (1), with a total of 58 enriched pathways. The top 20 enriched pathways (Figure 7f) were mainly related to the biosynthesis and degradation of terpenoids, alkaloids, fatty acids, carbohydrates, amino acids, and peptides. Given that terpenoids and alkaloids are commonly associated with antimicrobial activity, the enrichment of such pathways may reflect the metabolic responses of soil under long-term continuous cropping pressure and could selectively affect the structure of fungal communities.

### 3.7. Analysis of Key Differential Metabolite Expression Between Different Groups

**Figure 8:**
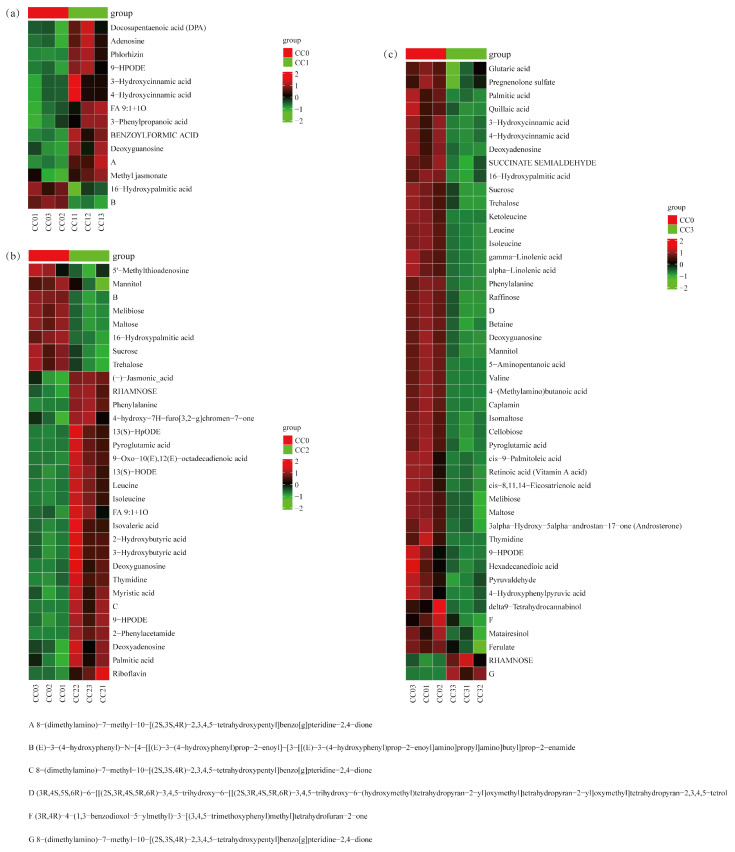
Heatmap of critical differential metabolites clustering enriched in the first five pathways among treatment groups. (**a**) CC0 vs. CC1, (**b**) CC0 vs. CC2, and (**c**) CC0 vs. CC3. CC0: Uncultivated; CC1: one cropping cycle; CC2: two cropping cycle; CC3: three cropping cycle.

According to the enrichment analysis results, key metabolic pathways closely associated with continuous cropping obstacles (CCOs) were further screened in each comparison group. In the CC0 vs. CC1 group (Figure 8a, Tables S4 and S5), a total of 14 differential metabolites were identified, which were significantly enriched in five KEGG pathways, including phenylalanine metabolism and degradation of aromatic compounds. Notably, five shikimic acid- and phenylpropanoid-derived compounds (3-hydroxycinnamic acid, 4-hydroxycinnamic acid, and 3-phenylpropanoic acid) were significantly upregulated and are known to possess potential autotoxicity. Two alkaloids exhibited opposing trends: 8-(dimethylamino)-7-methyl-benzo[g]pteridine-2,4-dione was significantly increased, while a complex amide-type phenylpropanoid derivative was significantly decreased. Additionally, four fatty acids (such as docosapentaenoic acid (DPA), methyl jasmonate, and 9-HPODE) and two carbohydrates (deoxyguanosine and adenosine) were markedly elevated, which are potentially involved in the early allelopathic stress response.

In the CC0 vs. CC2 group (Figure 8b, Tables S4 and S5), 31 key differential metabolites were identified, mainly enriched in ABC transporters, phosphotransferase system (PTS), and biosynthetic pathways. Two alkaloids (riboflavin and 2-phenylacetamide) were significantly upregulated, some of which have been reported to possess allelopathic activity. One shikimic acid-derivative compound (4-hydroxy-7H-furo[3,2-g]chromen-7-one) also showed significant upregulation. Several fatty acids (e.g., 13(S)-HODE, (-)-jasmonic acid, and FA 9:1 + 1O) were significantly increased, while 16-hydroxypalmitic acid decreased. Several carbohydrates, including deoxyadenosine and thymidine, were elevated, whereas six sugars (e.g., sucrose and trehalose) were significantly reduced. Additionally, four amino acids (e.g., leucine and phenylalanine) were upregulated, suggesting enhanced nitrogen metabolism.

In the CC0 vs. CC3 group (Figure 8c, Tables S4 and S5), 46 significant differential metabolites were detected, primarily enriched in pathways related to amino acid biosynthesis, starch and sucrose metabolism, and ABC transporters. Six shikimic acid- and phenylpropanoid-related compounds (such as ferulate and hydroxycinnamic acid derivatives) were significantly downregulated, suggesting their accumulation may occur at earlier stages and contribute to autotoxic effects. One alkaloid, 8-(dimethylamino)-7-methyl-benzo[g]pteridine-2,4-dione, was significantly upregulated. Several fatty acids (e.g., gamma-linolenic acid and glutaric acid) and carbohydrates (e.g., sucrose and cellobiose) were decreased, whereas RHAMNOSE was significantly increased. Furthermore, seven amino acids (e.g., leucine, valine, and betaine) and five terpenoids (e.g., quillaic acid and retinoic acid (vitamin A acid)) showed significant downregulation, indicating that both nutrient and secondary metabolism were suppressed under high continuous cropping stress.

### 3.8. Correlation Analysis of Differential Metabolites and Microbial Communities

**Figure 9:**
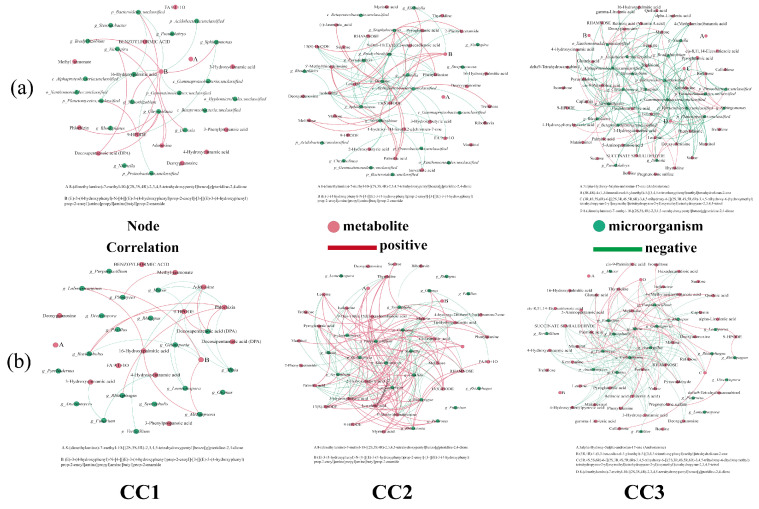
Correlation of the top 20 (**a**) bacteria and (**b**) fungi, in terms of relative abundance, with the differentially expressed metabolites involved in metabolic pathways. Red dots: metabolites; green dots: microorganisms; red line: positive correlation; green line: negative correlation. CC0: Uncultivated; CC1: one cropping cycle; CC2: two cropping cycle; CC3: three cropping cycle.

To further explore the relationship between soil metabolites and rhizosphere microbial communities under continuous cropping, Spearman correlation analysis (|r| > 0.8, *p* < 0.05) was performed to construct networks between key differential metabolites and the top 20 most abundant bacterial and fungal genera in each treatment group (Figure 9).

In the bacterial community (Figure 9a, Table S5), significant correlations were observed between several genera and differential metabolites across all comparisons. In the CC1 group, 11 bacterial genera showed strong correlations with six metabolites. For instance, *Sphingomonas*, enriched in CC1, was negatively correlated with the lipoxygenase product 9-HPODE (r = –0.9429) and positively correlated with a phenylpropanoid amide derivative (r = 0.8857). *Nitrospira* was positively associated with docosapentaenoic acid (DPA) and adenosine. *Chryseolinea* showed strong positive correlations with 9-HPODE, DPA, adenosine, and phlorhizin. In the CC2 group, 25 metabolites were significantly correlated with 12 bacterial genera. For example, *Pseudolabrys* was positively correlated with palmitic acid and 5′-methylthioadenosine, but negatively correlated with several metabolites, including 13(S)-HODE, pyroglutamic acid, leucine, isoleucine, deoxyguanosine, and phenylalanine. *Bradyrhizobium* exhibited negative correlations with 13(S)-HODE, 9-oxo-10(E),12(E)-octadecadienoic acid, phenylalanine, and 2-phenylacetamide. In the CC3 group, 39 metabolites were significantly correlated with 20 bacterial genera. *Nitrospira* showed negative correlations with reducing sugars (melibiose and maltose) and alkaloids, but was positively correlated with thymidine. *Mesorhizobium* was negatively associated with hexadecanedioic acid and positively with deoxyguanosine. Overall, beneficial bacteria such as *Sphingomonas*, *Bradyrhizobium*, *Devosia*, *Nitrospira*, and *Pseudolabrys* exhibited negative correlations with many upregulated metabolites (e.g., fatty acids, phenylpropanoids, alkaloids, and amino acids), and their relative abundance declined with continuous cropping years. These findings suggest that metabolite accumulation in continuously cropped soils may inhibit beneficial bacteria through nutrient competition or autotoxic effects.

In the fungal community (Figure 9b, Table S6), eight metabolites were significantly associated with 12 fungal genera in the CC1 group. For instance, *Mucor* was negatively correlated with 9-HPODE, adenosine, and phlorhizin. *Fusarium* showed negative associations with 4-hydroxycinnamic acid and 3-hydroxycinnamic acid, while *Verticillium* was negatively correlated with adenosine. In the CC2 group, 30 metabolites were significantly correlated with 15 fungal genera. Sugars such as melibiose and maltose were positively associated with the symbiotic fungus *Glomus*, while FA 9:1 + 1O was negatively correlated. *Purpureocillium* showed positive correlations with several fatty acids but negative correlations with unsaturated fatty acids and organic acids. *Rhizoctonia* was positively correlated with amino acids and rhamnose, but negatively associated with sugars like melibiose and maltose. In the CC3 group, 37 metabolites were significantly correlated with 18 fungal genera. Pathogenic *Aspergillus* showed negative correlations with multiple metabolites, including ketoleucine, quillaic acid, mannitol, amino acids, and nucleosides. *Fusarium* was positively correlated with ferulate and several sugars. *Rhizopus* and *Verticillium* were associated with specific nucleosides and fatty acids. Overall, pathogenic fungi were positively associated with allelopathic compounds such as fatty acids and terpenoids, while beneficial fungi like *Glomus* and *Purpureocillium* were negatively associated. These results suggest that metabolite accumulation under continuous cropping promotes the proliferation of pathogens and suppresses beneficial fungi, thereby contributing to soil microecological imbalance.

## 4. Discussion

In agricultural production, succession barriers caused by continuous cropping represent a major challenge to sustainable farming systems. Long-term monoculture significantly alters the physicochemical properties of soil, reduces microbial diversity, and leads to the accumulation of allelopathic compounds in the rhizosphere. In the case of *Dictyophora rubrovolvata*, continuous cultivation has been reported to increase soil acidity in the rhizosphere, thereby reducing yield [9]. This phenomenon is often accompanied by decreased levels of AN, AP, and AK. The present study observed consistent patterns, including significant reductions in soil pH, AP, and TK, along with declines in OM and TP. In contrast, the levels of AN, AK, and TN showed an increasing trend.

### 4.1. Microbial Community Shifts Induced by Continuous Cropping of Dictyophora rubrovolvata

Continuous cropping of *Dictyophora rubrovalvata* markedly altered the diversity and composition of the soil microbial community. Fungal diversity, as indicated by the Shannon and Simpson indices, progressively increased with cropping years, whereas bacterial diversity remained relatively stable. These findings differ from prior reports that documented a reduction in bacterial diversity and a nonsignificant increase in fungal diversity under similar conditions [41], possibly due to differences in fungal strains, cultivation periods, or environmental conditions. Similar patterns have been observed in other crops. For example, in cut-flower chrysanthemum, continuous cropping had minimal impact on bacterial communities but significantly increased fungal diversity, leading to a greater pathogen prevalence and disease incidence [42].

At the phylum level, continuous cropping led to declines in *Proteobacteria* and *Bacteroidetes*—key taxa involved in nutrient cycling and metabolic activity—while increasing the abundance of *Acidobacteria*, often associated with acidic and nutrient-limited soils [43,44,45]. This shift may be attributed to the accumulation of organic acids during cultivation, which exacerbates soil acidification. *Actinobacteria*, known for antibiotic production and disease suppression, also declined significantly under continuous cropping, indicating a possible loss of natural pathogen resistance [46,47]. At the genus level, notable reductions were observed in beneficial bacteria such as *Sphingomonas*, *Bradyrhizobium*, *Devosia*, *Nitrospira*, and *Pseudolabrys*. Similar trends have been documented in *Ganoderma leucocontextum* cultivation systems, supporting the conclusion that prolonged monoculture suppresses beneficial microbial taxa [48,49].

Fungal community structure also shifted, with an increasing dominance of *Ascomycota*—home to many phytopathogenic fungi—despite *Basidiomycota* remaining prevalent due to its saprotrophic capacity [50,51]. Genera such as *Fusarium*, *Rhizoctonia*, and *Aspergillus* became enriched with successive cropping years [52,53,54], while beneficial fungi including *Kluyveromyces*, *Diversispora*, and *Glomus* declined. These beneficial taxa are known to enhance nutrient uptake, trigger plant resistance, and function as biocontrol agents [55,56,57]. Overall, continuous cropping tends to promote pathogenic fungal enrichment and suppress beneficial microbes, disrupting microbial balance and increasing disease risk.

### 4.2. Alterations in Soil Metabolic Pathways Induced by Continuous Cropping

Soil metabolites, derived from root exudates, microbial activity, and organic matter decomposition, are central to plant–soil interactions [58,59]. Under continuous cropping stress, plants often alter root secretions by increasing allelopathic substances, which are recognized as contributors to replant disorders [60,61,62].

In the CC1 group, metabolite profiles differed significantly from those in CC0. KEGG analysis revealed enrichment in phenylalanine metabolism, purine metabolism, aromatic compound degradation, and secondary metabolite biosynthesis pathways. Multiple compounds with known autotoxic effects—including cinnamic acid derivatives, jasmonic acid, chalcones, and pteridine alkaloids—were found at elevated levels [63,64,65,66]. Jasmonic acid, for instance, is a stress-responsive hormone known to inhibit growth and contribute to rhizosphere degradation under continuous cropping conditions.

In the CC2 group, pathways related to ABC transporters, aminoacyl-tRNA biosynthesis, and PTS systems were enriched. Accumulated metabolites included myristic acid, fatty acids, alkaloids, phenolic acids, and coumarins—many of which exhibit concentration-dependent dual roles as signaling molecules and inhibitors, demonstrating the “low-dose stimulation, high-dose inhibition” effect [67,68,69]. Myristic acid, in particular, has been associated with pathogen enrichment in long-term tobacco monoculture systems [70].

In the CC3 group, biosynthesis of amino acids, carbohydrate metabolism, and transporter activity remained pronounced. Monosaccharide accumulation coincided with declines in branched-chain fatty acids and amino acids. The presence of elevated glycerophospholipids and esterified fatty acids suggests that organic compound accumulation may contribute to the formation of cropping barriers [71].

These results indicate that continuous cropping drives the accumulation of allelopathic metabolites, which likely impair soil quality and inhibit plant performance.

### 4.3. Correlations Between Mycelial Metabolites and Microbial Communities

Spearman correlation analysis (|r| > 0.8, *p* < 0.05) revealed strong associations between specific metabolites and microbial genera. Pathogenic fungi such as *Mucor*, *Fusarium*, *Verticillium*, and *Aspergillus* showed significant positive correlations with compounds like palmitic acid, jasmonic acid, ferulic acid, and pseudoalkaloids—all previously implicated in autotoxic effects [72,73,74,75].

Conversely, beneficial fungi including *Glomus* and *Purpureocillium* exhibited negative correlations with the same metabolites. As an arbuscular mycorrhizal fungus (AMF), *Glomus* showed inverse relationships with palmitic acid, phenylpropanoids, and fatty acids, suggesting that allelopathic accumulation may suppress symbiotic fungi. Similarly, beneficial bacterial genera such as *Bradyrhizobium*, *Devosia*, and *Sphingomonas* also showed negative correlations with allelopathic substances, consistent with previous findings that root-secreted metabolites can selectively inhibit beneficial microbes while enhancing pathogen proliferation [76].

Taken together, the accumulation of autotoxic metabolites under continuous cropping appears to favor pathogenic microbes and inhibit beneficial ones, disrupting microbial homeostasis and potentially driving soilborne disease outbreaks.

### 4.4. Study Limitations and Practical Implications

Although this study provides meaningful insights into the microbial and metabolic responses to continuous cropping of *Dictyophora rubrovalvata*, certain limitations should be acknowledged. First, the correlations between key metabolites and microbial genera were derived from statistical analysis, and causality remains unproven. Functional validation through targeted experiments, such as microbial inoculation or metabolite addition, is needed to clarify these relationships. Second, the investigation was restricted to a single geographic site, which may limit the generalizability of the findings. Broader regional sampling would help address environmental variability. Third, while multiple allelopathic compounds were identified, their specific physiological impacts on microbial activity and plant–microbe interactions remain to be fully elucidated.

To address the continuous cropping obstacles, several integrative strategies are recommended. Diversified planting systems, such as crop rotation and intercropping, can improve soil biodiversity, disrupt pathogen transmission, and reduce allelochemical accumulation. In particular, rotation with non-host crops has been shown to enhance soil structure and nutrient cycling while suppressing soilborne pathogens. The balanced application of macro and micronutrients, along with organic amendments or biochar, helps optimize soil physicochemical conditions, support microbial diversity, and mitigate acidification. Additionally, the inoculation of beneficial microorganisms—such as *Sphingomonas*, *Bacillus*, and arbuscular mycorrhizal fungi like *Glomus*—has been reported to improve microbial community balance, suppress pathogens, and promote plant health.

While each approach has its limitations, their combined application offers a sustainable and ecologically sound strategy for overcoming continuous cropping barriers in *Dictyophora rubrovalvata* cultivation.

## 5. Conclusions

This study systematically investigated the effects of continuous cropping on soil physicochemical properties, microbial communities, and metabolic profiles in *Dictyophora rubrovalvata*-cultivated soils. The results revealed that continuous cropping significantly altered soil conditions, disrupted microbial balance, and promoted the accumulation of allelopathic metabolites, particularly those associated with pathogenic fungi enrichment and the decline of beneficial microbes. Integrated metagenomic and metabolomic analyses further demonstrated strong correlations between key autotoxic compounds (e.g., ferulic acid and palmitic acid) and microbial community shifts, suggesting a feedback mechanism contributing to continuous cropping obstacles.

These findings provide important insights into the mechanisms underlying soil degradation and microbial dysbiosis under monoculture conditions. Future studies should prioritize functional validation of candidate metabolites and microbes and expand field experiments across multiple ecological regions to develop more generalizable and sustainable cultivation strategies for *Dictyophora rubrovalvata*.

## Figures and Tables

**Table 1:** Soil physicochemical properties under continuous cropping of *Dictyophora rubrovolvata*.

Factor	CC0	CC1	CC2	CC3
pH	7.94 ± 0.07 a	7.90 ± 0.07 a	7.60 ± 0.09 b	7.52 ± 0.07 b
OM(g/kg)	235.26 ± 25.72 a	222.77 ± 56.08 a	230.67 ± 12.66 a	208.33 ± 14.57 a
AN(mg/kg)	284.5 ± 45.91 c	330.08 ± 16.09 c	1055 ± 51.51 a	886.33 ± 57 b
AP(mg/kg)	213.69 ± 4.62 a	184.58 ± 5.12 b	35.87 ± 17.81 c	15.7 ± 4.65 c
AK(mg/kg)	484.31 ± 21.81 b	465.41 ± 113.69 b	525.67 ± 27.39 b	693 ± 51.22 a
TN(g/kg)	7.7 ± 0.85 a	8.32 ± 0.44 a	9.47 ± 0.42 a	9.24 ± 1.19 a
TP(g/kg)	1.51 ± 0.25 a	1.52 ± 0.04 a	1.15 ± 0.04 a	1.05 ± 0.38 a
TK(g/kg)	22.63 ± 1.35 a	22.29 ± 2.45 a	5.27 ± 0.21 b	4.48 ± 0.77 b

Note: Different letters within the same row indicate significant differences among treatments (*p* < 0.05, ANOVA, Tukey-HSD test). pH; OM (organic matter); AN (alkali-hydrolyzable nitrogen); AP (available phosphorus); AK (available potassium); TN (total nitrogen); TP (total phosphorus); TK (total potassium). CC0: Uncultivated; CC1: one cropping cycle; CC2: two cropping cycle; CC3: three cropping cycle.

## Data Availability

The raw sequencing data have been deposited in the NCBI Sequence Read Archive (SRA) under the accession number PRJNA1255718 (https://www.ncbi.nlm.nih.gov/sra/PRJNA1255718, accessed on 26 April 2025). Other data supporting the findings of this study are available from the corresponding author upon reasonable request.

## Supplementary Materials

The following supporting information can be downloaded at https://www.mdpi.com/article/10.3390/microorganisms13092186/s1, Table S1: Experimental design; Table S2: Temperature, humidity and light control parameters at different developmental stages of *Dictyophora rubrovalvata*; Table S3: LDA scores of microbial biomarkers identified by LEfSe under different continuous cropping treatments; Table S4: KEGG pathway enrichment analysis of differential metabolites; Table S5: Differential metabolite expression enriched in the top 5 KEGG pathways among different comparison groups; Table S6: Correlations between bacteria and differential metabolites; Table S7: Correlations between fungi and differential metabolites; Figure S1: Indicator bacteria (LDA > 3); Figure S2: Indicator fungi (LDA > 3).

## Abbreviations

The following abbreviations are used in this manuscript:

PCoA	Principal Coordinates Analysis
LEfSe	Linear Discriminant Analysis Effect Size
PCA	Principal Components Analysis
OPLS-DA	Orthogonal Partial Least Squares—Discriminant Analysis
UHPLC–OE–MS	Ultra-High Performance Liquid Chromatography–Orbitrap Exploris Mass Spectrometry
