# Supplementary material for: Elucidation of Mechanism of Soil Degradation Caused by Continuous Cropping of Dictyophora rubrovalvata Using Metagenomic and Metabolomic Technologies
Source: Microorganisms. 2025 Sep 19;13(9):2186. doi: 10.3390/microorganisms13092186 (PMC12473074; doi:10.3390/microorganisms13092186)
Supplement: Supplementary file 1 [file microorganisms-13-02186-s001.zip › Figures S1 and S2.pdf]

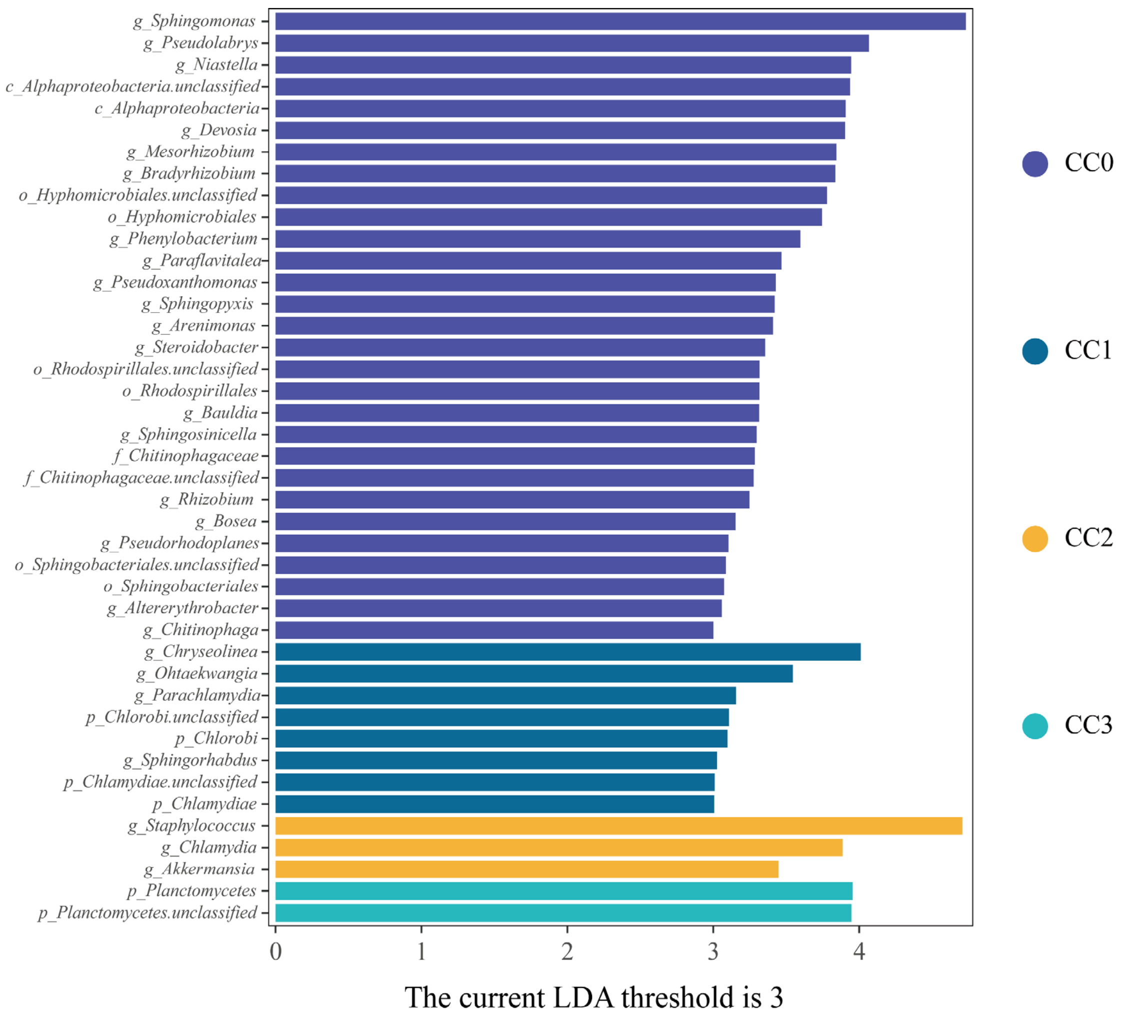

Figure S1. Indicator bacteria (LDA > 3). p: phylum, o: order, c: class, f: family, g: genus. CC0: Uncultivated; CC1: one cropping cycle; CC2: two cropping cycle; CC3: three cropping cycle.

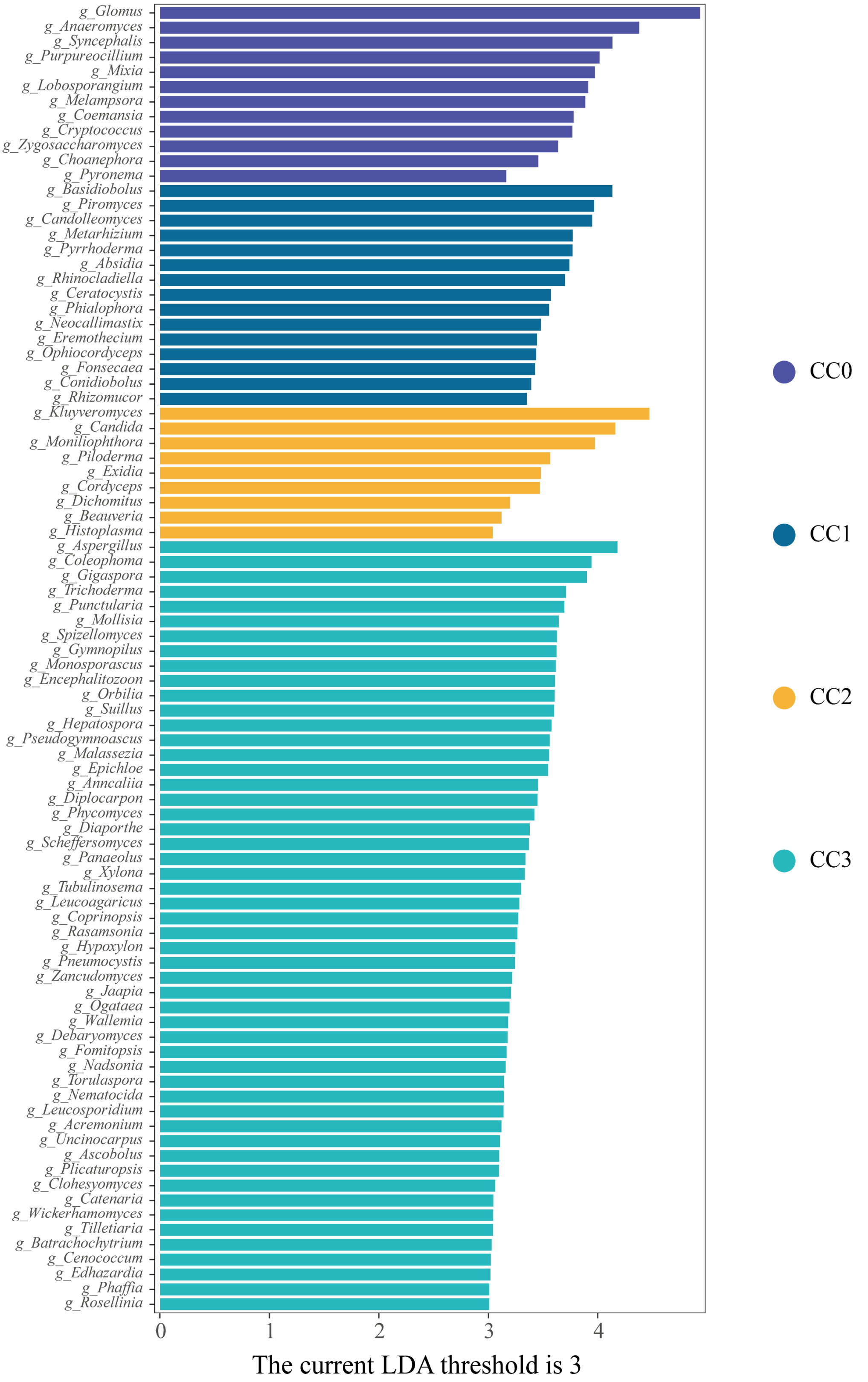

Figure S2. Indicator fungi (LDA > 3). p: phylum, o: order, c: class, f: family, g: genus. CC0: Uncultivated; CC1: one cropping cycle; CC2: two cropping cycle; CC3: three cropping cycle.
